# Low production of reactive oxygen species in granulocytes is associated with organ damage in systemic lupus erythematosus

**DOI:** 10.1186/ar4575

**Published:** 2014-06-05

**Authors:** Anders A Bengtsson, Åsa Pettersson, Stina Wichert, Birgitta Gullstrand, Markus Hansson, Thomas Hellmark, Åsa CM Johansson

**Affiliations:** 1 Department of Clinical Sciences, Section of Rheumatology, Lund University and Skåne University Hospital, 221 85 Lund, Sweden; 2 Department of Nephrology, Clinical Sciences in Lund, Lund University, BMC B13, 221 84 Lund, Sweden; 3 Department of Haematology, Lund University, BMC B13, 221 84 Lund, Sweden; 4 Department of Laboratory Medicine Lund. Section of Microbiology, Immunology and Glycobiology, Lund University, Lund, Sweden; 5 Clinical Immunology and Transfusion Medicine, University and Regional Laboratories Region Skåne, 221 85 Lund, Sweden

## Abstract

**Introduction:**

Polymorphonuclear leukocytes (PMN) are main effector cells in the acute immune response. While the specific role of PMN in systemic lupus erythematosus (SLE) and autoimmunity is still unclear, their importance in chronic inflammation is gaining more attention. Here we investigate aspects of function, bone marrow release and activation of PMN in patients with SLE.

**Methods:**

The following PMN functions and subsets were evaluated using flow cytometry; (a) production of reactive oxygen species (ROS) after *ex vivo* stimulation with phorbol 12-myristate 13-acetate (PMA) or *Escherichia coli (E. coli)*; (b) capacity to phagocytose antibody-coated necrotic cell material; (c) PMN recently released from bone marrow, defined as percentage of CD10^−^D16^low^ in peripheral blood, and (d) PMN activation markers; CD11b, CD62L and C5aR.

**Results:**

SLE patients (n = 92) showed lower ROS production compared with healthy controls (n = 38) after activation *ex vivo*. The ROS production was not associated with corticosteroid dose or other immunotherapies. PMA induced ROS production was significantly reduced in patients with severe disease. In contrast, neither ROS levels after *E. coli* activation, nor the capacity to phagocytose were associated with disease severity. This suggests that decreased ROS production after PMA activation is a sign of changed PMN behaviour rather than generally impaired functions. The CD10^−^CD16^low^ phenotype constitute 2% of PMN in peripheral blood of SLE patients compared with 6.4% in controls, indicating a decreased release of PMN from the bone marrow in SLE. A decreased expression of C5aR on PMN was observed in SLE patients, pointing towards *in vivo* activation.

**Conclusions:**

Our results indicate that PMN from SLE patients have altered function, are partly activated and are released abnormally from bone marrow. The association between low ROS formation in PMN and disease severity is consistent with findings in other autoimmune diseases and might be considered as a risk factor.

## Introduction

Systemic lupus erythematosus (SLE) is a chronic systemic autoimmune disease affecting several organ systems such as skin, joints, kidneys and central nervous system. Many of the disease manifestations in SLE are related to immune complexes, consisting of autoantibodies and remnants of apoptotic cells [1]. Apoptotic cells are thought to be a major source of auto-antigens in SLE, partly because of impaired clearance [2,3]. Another potential antigen source is the neutrophil extracellular traps (NETs) that consist of chromatin and antimicrobial enzymes released from neutrophils to trap and kill pathogens. Serum from some SLE patients have a reduced ability to degrade NETs [4,5].

Polymorphonuclear leukocytes (PMN), such as neutrophils, are produced in the bone marrow and released to circulation. During acute inflammation an increased mobilization of neutrophils from the bone marrow occurs, which can be observed as increased percentage of CD10^−^CD16^low^ neutrophils in peripheral blood [6,7]. The role of PMN in chronic inflammation and autoimmunity is coming into focus, and neutrophils have been suggested to be the primary mediators of end organ-damage responding to deposited immune complexes [8,9]. PMN are recruited to inflammatory sites, and activated by pro-inflammatory mediators like complement factors, cytokines and chemokines. Upon activation the expression of various surface proteins changes; for example, C5aR and CD62L are down regulated whereas an increase in CD11b expression is observed [10,11]. In addition to the changing expression of surface proteins, activated PMN are primed to release granules and produce reactive oxygen species (ROS) by the nicotinamide adenine dinucleotide phosphate-oxidase (NADPH) complex [12]. ROS are major effector molecules in inflammatory processes and tightly linked to NETs formation. During the last decade, an increasing amount of data support a T-cell regulating role for monocyte and PMN-produced ROS [13]–[16]. Furthermore, the association of SLE to polymorphism in *NCF2*, encoding a protein in the NADPH oxidase complex, adds support for the importance of ROS in this disease [17]. Of note, patients with chronic granulomatous disease, lacking a functional NADPH oxidase complex, show autoimmune features such as high levels of immunoglobulins and autoantibodies, as well as an increased risk of Crohn’s disease and discoid lupus [18,19].

This study aims at characterizing PMN from SLE patients (SLE-PMN), in regard to function, bone marrow release and activation to gain knowledge of the role of PMN in SLE and autoimmunity.

## Methods

### Patients and controls

SLE patients (n = 107) were recruited to the study, when coming to their scheduled visit at the Department of Rheumatology, Skåne University Hospital, Lund, Sweden. All patients fulfilled at least four American College of Rheumatology classification criteria for SLE [20]. Disease activity was assessed using the systemic lupus erythematosus disease activity index 2000 (SLEDAI-2 K) [21], and organ damage was evaluated according to the Systemic Lupus International Collaborative Clinics/American College of Rheumatology damage index (SLICC/ACR-DI) [22]. Demographic and clinical characteristics are shown in Table 1. Healthy blood donors (n = 38, Blood centre in Lund) and healthy volunteers (n = 15) were recruited as controls; ages 18 to 65 years. Complement proteins and autoantibodies were measured using routine analyses (Clinical Immunology and Transfusion Medicine, University and Regional laboratories, Region Skåne, Lund Sweden). The study was approved by the Regional Ethics Review Board at Lund University (file number LU 2010-708) and informed consent was obtained from all participants.

**Table 1:** Patients characteristics and demographics

	**All patients (n = 92)**	**No organ damage SLICC/ACR-DI = 0 (n = 42)**	**Organ damage SLICC/ACR-DI ≥1 (n = 50)**
Age, median (range) years	48 (22 to 84)	43 (22 to 79)	60 (24 to 84)
Female gender, n (%)	81 (88%)	37 (88%)	44 (88%)
Disease duration, median (range) years	14 (0 to 51)	9 (0 to 29)	19 (0 to 51)
SLEDAI, median (range)	2 (0 to 16)	2 (0 to 16)	1 (0 to 13)
SLICC/ACR-DI median (range)	1 (0 to 8)	0	2 (1 to 8)
PMN 10^9^/L median (range)	4.0 (<0.1 to 11)	3.6 (1.4 to 9.8)	4.7 (<0.1 to 11)
**Disease manifestations at time of sampling, n**			
Lupus headache	1	0	1
Arthritis	10	5	5
Kidney involvement (urinary cast, hematuria, proteinuria, or pyuria)	6	3	3
Rash	4	2	2
Alopecia	2	1	1
Low complement (C3 or C4)	36	21	15
Anti-double stranded DNA antibodies	18	10	8
Leukopenia	7	4	3
**Treatment**			
Prednisone, % (median dose of treated patients)	53 (5 mg)	60 (5 mg)	50 (6.25 mg)
Hydroxychloroquine, % (n)	59 (54)	69 (29)	50 (25)
Chloroquine phosphate, % (n)	2 (2)	2 (1)	2 (1)
Azathioprine, % (n)	24 (22)	29 (12)	20 (10)
Mycophenolate mofetil%, (n)	12 (11)	14 (6)	10 (5)
Rituximab, % (n)	2 (2)	0 (0)	4 (2)
Methotrexates, % (n)	4 (4)	5 (2)	4 (2)
Cyclosporine A, % (n)	2 (2)	0 (0)	4 (2)

SLICC/ACR-DI, Systemic Lupus International Collaborative Clinics/American College of Rheumatology (ACR) damage index; SLEDAI-2 K, Systemic lupus erythematosus disease activity index 2000; PMN, polymorphonuclear leukocytes.

### Oxidative burst and expression of surface markers

ROS production in peripheral blood PMN was investigated using the PhagoBurst assay, Glycotope Biotechnology, GmBH, Germany, according to the manufacturer’s protocol after activation with phorbol 12-myristate 13-acetate (PMA) or opsonised *Escherichia coli* (*E. coli*), and analysed using flow cytometry. At least 15,000 PMN were analysed based on forward and side scatter properties. No patient with ROS deficiency was observed.

ROS formation in peripheral blood PMN was also quantified by oxidation of 2,7-dichlorofluorescein-diacetate (DCFH-DA, Sigma-Aldrich®, St. Louis, MO, USA), as previously described [23]. As stimuli PMA and *E. coli* from the PhagoBurst kit or *Staphylococcus aureus* (ATCC 25923, 1 leukocyte: 2,000 bacterial cells) and *Pseudomonas aeruginosa* (ATCC 27853, 1 leukocyte: 200 bacterial cells) were used. *S. aureus* and *P. aeruginosa* were grown in liquid Tryptic Soy Broth (TSB) medium overnight at 37°C and killed by heat (60°C) for 2 h. To confirm bacterial inactivation a sample was inoculated in TSB and kept for 48 h. The bacteria were centrifuged and re-suspended in 0.8% saline. Optical density was adjusted to 24 × 10^8^ colony forming units/mL by comparing turbidity to a McFarland scale number 8 BaSO_4_ standard solution. DCFH-DA was added to heparinised whole blood before the various stimuli, and then the samples were incubated in a 37°C water bath for 30 minutes. Cells were analysed using flow cytometry.

The expression of selected surface markers on PMN was analysed using flow cytometry. Briefly, peripheral blood was lysed using 0.84% ammonium chloride. The remaining leukocytes were stained for surface expression of CD14 (to exclude monocytes), CD10, CD11b, CD16, CD62L, and C5aR (CD88) (BD Bioscience San Jose, CA, USA). For flow cytometry analysis a FACSCanto II and the DIVA software (Becton Dickinson, BD, New York, NY, USA) were used.

### Cell separation and phagocytosis of antibody-coated necrotic cell material by PMN

PMN and peripheral blood mononuclear cells were isolated from heparinised blood of SLE patients by density gradient centrifugation on Polymorphprep™ (Axis-Shield Poc AS, Norway). To obtain necrotic cell material, mononuclear cells were incubated for 10 minutes at 70°C and stained with propidium iodide (BD Bioscience). The propidium iodide-labelled necrotic cell material (4.5 × 10^5^ cells) was then incubated with or without an anti-nucleosome antibody (clone PL2-3; gift from Marc Monestier, Temple University, Philadelphia, USA) at room temperature for 20 minutes. Normal human serum was used as the negative control. The autologous PMN were stained with anti-CD45-FITC (BD Bioscience), and then added to the necrotic cell material, at a concentration of 1.0 × 10^6^ cells/mL in a total volume of 300 μL, followed by incubation at 37°C for 15 minutes. Cells were washed with phosphate-buffered saline pH 7.2 containing 0.1% human serum albumin (Sigma-Aldrich, St. Louis, MO, USA) before analysis by flow cytometry.

### Statistical analysis

Correlations were determined by Spearman’s correlation test. The Mann-Whitney *U*-test was used for two-group comparisons and Kruskal-Wallis test with Dunn’s multiple comparison test was used for three-group comparisons. All *P*-values were considered significant at *P* <0.05.

## Results

### Decreased production of ROS in SLE-PMN

**Figure 1:**
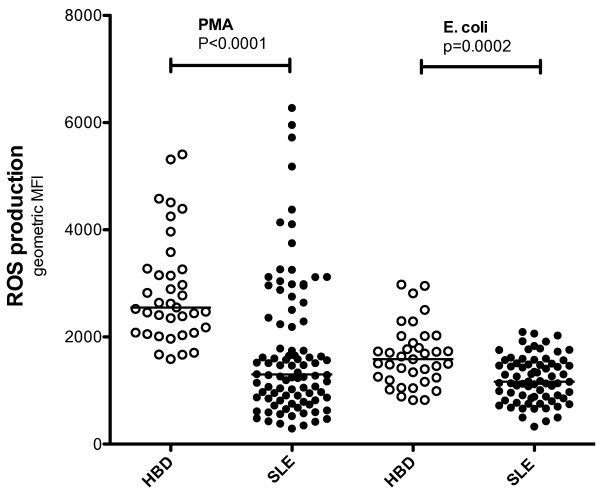
**Polymorphonuclear leukocytes (PMN) from patients with systemic lupus erythematosus (SLE) produced fewer reactive oxygen species (ROS) than PMN from healthy blood donors.** The capacities of PMN from healthy blood donors (HBD), n = 38, and SLE patients, n = 92, to produce ROS upon activation with phorbol 12-myristate 13-acetate or opsonised *E. coli* were investigated using flow cytometry. The amount of ROS produced is shown as geometric mean fluorescence intensity (geo mean fluorescence, MFI). The two-sided Mann-Whitney test was used to calculate the level of significance. The horizontal lines represent the median value of each dataset.

**Figure 2:**
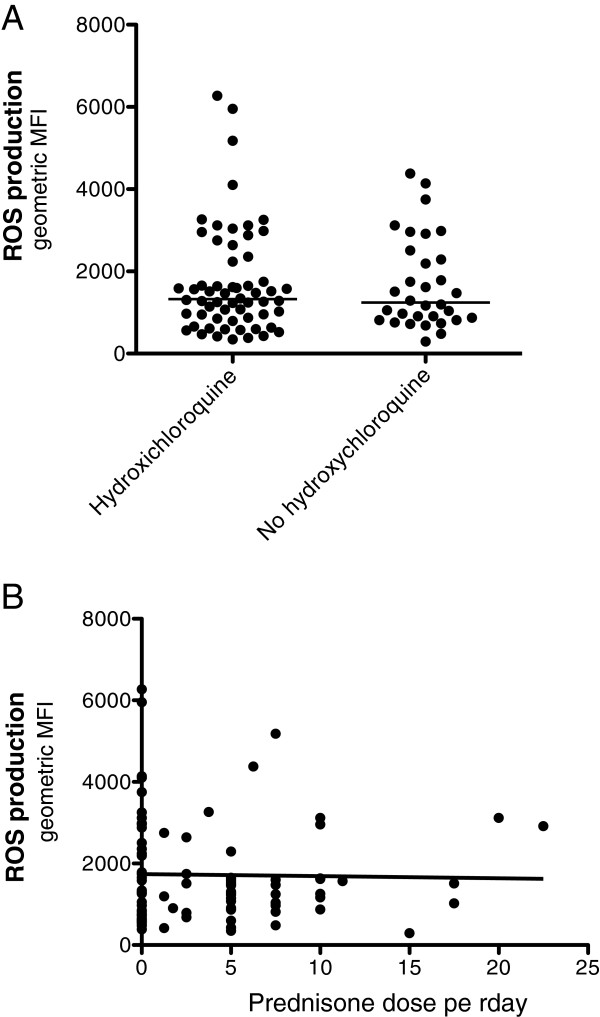
**Reactive oxygen species (ROS) production in polymorphonuclear leukocytes (PMN) from systemic lupus erythematosus (SLE) patients did not correlate with Hydroxychloroquine treatment or the dose of prednisone.** ROS production was measured by flow cytometry after *ex vivo* activation of PMN with phorbol 12-myristate 13-acetate **(A)** the amount ROS produced by SLE-PMN, treated with or without Hydroxychloroquine. **(B)** The amount of ROS produced in individual patients plotted against the dose of prednisone at the day of sampling. The amount ROS produced is shown as geometric mean fluorescence intensity (geo mean fluorescence, MFI). The line represents the median value of each dataset.

Phagocyte-produced ROS are important effector molecules in the defence against microbes, and could also be involved in the regulation of the adaptive immune system [15]. To evaluate PMN function in SLE, we decided to investigate intracellular ROS production. PMN in peripheral whole blood from SLE patients (n = 92, Table 1) and healthy controls (n = 38), were stimulated with either the protein kinase C activator, PMA, or with opsonised *E. coli*. SLE-PMN showed a decreased capacity to produce ROS *ex vivo* after activation with both PMA (*P* <0.0001) and *E. coli* (*P* = 0.0002) (Figure 1). The decreased amount of ROS produced by SLE-PMN was not associated with the dose of prednisone or hydroxychloroquine treatment (Figure 2A and B) or other immune suppressive drugs listed in Table 1 (not shown). Perazzio *et al*. have previously shown an increased ROS production in SLE-PMN after *in vitro* activation with *S. aureus* or *P. aeruginosa* using DCFH-DA as fluorochrome [23]. To evaluate whether this discrepancy was due to experimental procedure or differences in patient population, patients (n = 15) and controls (n = 15) were analysed in parallel with both methods, using *S. aureus, P. aeruginosa,* PMA and *E. coli* as stimuli. Similar findings where observed between the two methods (Table 2). SLE-PMN showed a decreased intracellular ROS formation after PMA activation compared with controls (PhagoBurst test: *P* = 0.0394 and DCFH-DA: *P* = 0.0146) whereas no significant difference was observed with the other stimuli (not shown). The decreased ROS production in our examined SLE cohort after PMA activation was consistent using both methods in contrast to the findings of Perazzio *et al*., suggesting differences in patient populations.

**Table 2:** Comparisons between the PhagoBurst and the DCFH-DA assay

	**Relative reactive oxygen species formation in SLE patients as% of formation in healthy controls**		
**Stimuli**	**PhagoBurst**	**DCFH-DA**	***P*-value**
PMA	68 ± 7.7	76 ± 6.2	0.6783
*E. coli*	84 ± 13	103 ± 13	0.1775
*S. aureus*	92 ± 8.7	102 ± 6.3	0.2716
*P. aeruginosa*	88 ± 12	97 ± 14	0.5897

Comparisons of the PhagoBurst assay with the dichlorodihydrofluorescein-diacetate (DCFH-DA) assay according to Perazzio *et al*. [23]. No significant differences between the methods were observed. Polymorphonuclear leukocytes (PMN) from systemic lupus erythematosus (SLE) patients (n = 15) and healthy controls (n = 15) were analysed in parallel with both methods using phorbol 12-myristate 13-acetate (PMA), *E. coli*, *S. aureus or P. aeruginosa* as stimuli*.* ROS formation was defined as geometric mean fluorescence intensity. Samples from each individual patient were divided by the mean value of the controls to gain the relative ROS formation in SLE-PMN as% of ROS produced in healthy controls. Values represent mean ± standard error of the mean. The two-sided Mann-Whitney test was used to calculate the level of significance.

### Organ damage was associated with low ROS production in SLE-PMN

**Figure 3:**
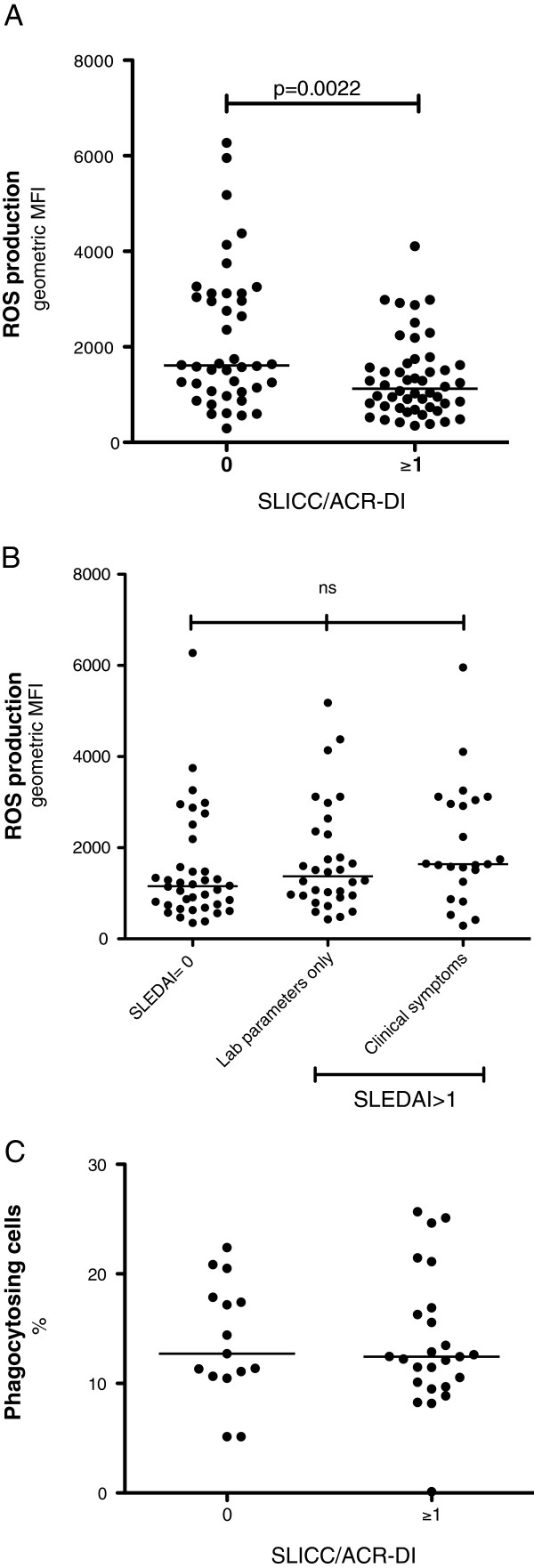
**Organ damage in systemic lupus erythematosus (SLE) patients is associated with decreased reactive oxygen species (ROS) production.** ROS production was measured by flow cytometry after *ex vivo* activation of peripheral blood polymorphonuclear leukocytes (PMN) with phorbol 12-myristate 13-acetate. **(A)** The amount of ROS produced by PMN from patients with organ damage (Systemic Lupus International Collaborative Clinics/ACR damage index (SLICC/ACR-DI) ≥1) was compared with PMN from patients without organ damage (SLICC/ACR-DI = 0). **(B)** The amount of ROS produced by PMN from patients with (1) inactive disease (SLE activity index 2000 (SLEDAI-2 K) = 0) affected laboratory parameters, such as low complement and anti-double stranded DNA antibodies, but no clinical symptoms; (2) laboratory parameters only, and patients with clinical manifestations, for example, nephritis, rash and arthritis; and (3) clinical symptoms (*P* = 0,0654). **(C)** Phagocytosis of necrotic cell material, in the presence of serum and anti-nucleosome antibodies, by purified polymorph nucleated leukocytes (n = 40), was analysed using flow cytometry. The patients were divided based on organ damage (SLICC/ACR-DI) and their phagocytosis capacity is shown as% phagocytosing cells. The two-sided Mann-Whitney test was used to calculate the level of significance between two groups and Kruskal-Wallis test with Dunn’s multiple comparison test was used to calculate the level of significance between three groups. The line represents the median value of each dataset. MFI, mean fluorescence intensity.

The severity of autoimmune diseases has previously been associated with decreased ROS production [24]–[26]. Hence, to study if the severity of SLE was associated with changes in ROS production, the patients were divided in two groups based on the presence of organ damage or not according to SLICC/ACR-DI (Table 1). PMN from patients with SLICC/ACR-DI ≥1 had decreased ROS production, compared with patients without organ damage (Figure 3A), when activated with PMA (*P* = 0.0022). No difference was seen after activation with *E. coli* (not shown). Patients with organ damage were in general older than patients without (Table 1), however, the age of the patients was not correlated with PMA-induced oxidative burst in PMN (not shown).

Next, we investigated if disease activity, at the time point of sampling, was associated with ROS production. The patients were divided into three groups based on the SLEDAI-2 K [21]: (1) no activity, (2) laboratory parameters only, such as low complement and anti-double stranded DNA antibodies; and (3) clinical manifestations, for example, nephritis, rash and arthritis. No association between ROS production and disease activity (*P* = 0.0654) was observed (Figure 3B).

Phagocytosis of antibody-coated microbes and foreign material precedes ROS production in PMN. To evaluate further the function of PMN in SLE, in particular in patients with organ damage, the phagocytosis capacity was investigated in 40 out of the 92 patients. Antibody-coated necrotic cells were chosen as stimuli for phagocytosis to relate to lupus erythematosus cells, for example, PMN containing phagocytosed antibody-coated dead cell materials, a phenomenon almost pathognomonic for SLE. No differences were observed between patients with (n = 25; SLICC/ACR-DI ≥1), compared to patients without organ damage (n = 15; SLICC/ACR-DI = 0), further suggesting that the decreased ROS production in patients with severe disease is not due to a general unresponsiveness (Figure 3C). No associations between disease activity based on SLEDAI-2 K and the ability to phagocytose were observed (not shown).

### Low numbers of CD10^−^CD16^low^ SLE-PMN

**Figure 4:**
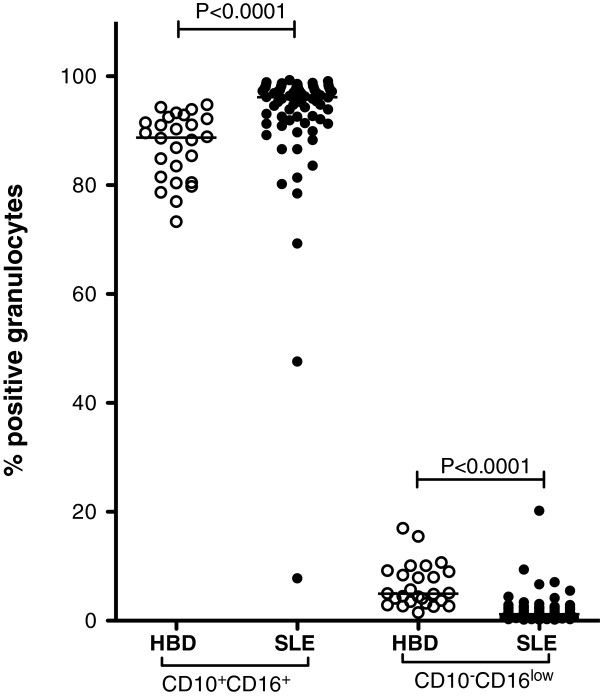
**Decreased numbers of CD10**^**−**^**CD16**^**low **^**polymorphonuclear leukocytes (PMN) from patients with systemic lupus erythematosus (SLE).** The frequencies of CD10^+^CD16^+^ (mainly segment nucleated neutrophils) and CD10^−^CD16^low^ (suggested as a marker for newly released neutrophils) PMN were investigated in healthy blood donors (HBD, n = 27) and patients (n = 73) using flow cytometry. The two-sided Mann-Whitney test was used to calculate the level of significance. The line represents the median value of each dataset.

During acute inflammation, an increased percentage of CD10^−^CD16^low^ neutrophils in peripheral blood are thought to reflect an increased mobilization of cells from bone marrow [6,7]. To study the frequency of newly released CD10^−^CD16^low^ PMN in peripheral blood, samples from 73 SLE patients, and 27 healthy controls were analysed by flow cytometry. SLE patients showed lower percentages of CD10^−^CD16^low^ PMN (Figure 4), compared with healthy controls (*P* <0.0001). Hence, the SLE-PMN were to a high extent CD10^+^CD16^+^ compared with controls (*P* <0.0001), which is consistent with a decreased release of PMN from the bone marrow.

To characterize the activation status of PMN in peripheral blood the expression of C5aR, CD11b and CD62L was investigated. SLE-PMN were to a lesser extent C5aR positive (*P* <0.0001), and the positive cells expressed less C5aR (*P* <0.0001) (Table 3). No differences in the expression of CD11b or CD62L were observed (Table 3), indicating that the cells were only partly activated. C5aR expression and percentage of CD10^−^CD16^low^PMN were not correlated with SLICC/ACR-DI or SLEDAI-2 K (not shown).

**Table 3:** Decreased expression of C5aR (CD88) on polymorphonuclear leukocytes (PMN) from patients with systemic lupus erythematosus (SLE)

**Phenotype**	**Healthy blood donors**	**SLE patients**	***P*-value**
CD88, %	92 ± 3.8	77 ± 1	≤0.0001
CD88, geoMFI	466 ± 24	285 ± 4	≤0.0001
CD62L, %	98 ± 0.3	98 ± 0.2	ns
CD62L, geoMFI	1180 ± 64	1100 ± 42	ns
CD11b, %	100 ± 0	100 ± 0	ns
CD11b, geoMFI	9343 ± 578	8166 ± 270	ns

Comparisons were done between SLE patients and healthy blood donors in regard of the frequency (%) and amount (geometric mean fluorescence intensity (geoMFI) of positive cells) of CD88, CD62L and CD11b on CD10^+^CD16^+^ PMN. Values represent mean ± standard error of the mean. The two-sided Mann-Whitney test was used to calculate the level of significance; ns, not significant.

## Discussion

PMN were characterized with respect to function, bone marrow release and activation to study their role in SLE, yielding evidence for decreased ROS production in SLE and autoimmunity. Our data support that SLE-PMN have decreased capacity to produce ROS *ex vivo.* The association with disease severity, defined as organ damage, further strengthened our finding. Low ROS production has been associated with disease severity of other autoimmune conditions, including Behcet’s disease [24], Guillain-Barre syndrome [25] and multiple sclerosis [26], and might be a common denominator important in the pathogenesis of autoimmunity.

Interestingly, PMA-induced ROS production was significantly reduced in patients with severe disease. However, neither ROS production after *E. coli* activation nor phagocytosis of necrotic cell material were associated with organ damage, suggesting that decreased ROS levels after PMA activation is not a sign of impaired PMN functions in general but rather a sign for changed PMN behaviour. While the activation and control of the NADPH oxidase in neutrophils (NOX2) is incompletely understood, it seems that different agonists encountered by the neutrophils engage various combinations of kinases and thereby affect the degree of activity of the NADPH complex, and in the end the amount of ROS produced [27]. To some extent, this could explain why ROS production after *E. coli* activation was not associated with organ damage; *E. coli* induced a lower degree of phosphorylation of the NADPH complex regulating subunits compared with PMA that is known to push the NADPH complex to its maximal capacity [27]. Hence, PMA revealed altered behaviour in PMN from patients with organ damage.

While no association between ROS levels and current disease activity was observed, most patients were in remission or had low to moderate activity based on SLEDAI-2 K (Table 1). An association between disease activity and ROS production could not be excluded based on the available data. The literature is not concurrent regarding ROS production by SLE-PMN [23,28,29]. For example, Perazzio *et al*. have shown that neutrophils from SLE patients have an increased capacity to produce ROS, and they did not find any correlation with organ damage or disease activity [23]. This discrepancy does not reflect the use of different methods, as we observed comparable results with both methods. A more likely explanation is variations in patient cohorts. We have observed an association between decreased ROS formation and disease severity, and a tendency towards increased ROS formation in SLE-PMN in patients with clinical symptoms. Most patients in our study were in remission and possibly our cohort contained more patients with organ damage giving rise to the divergent results. In addition, an influence of genetic factors could not be excluded.

Corticosteroids have been reported to affect the ROS production in PMN in a cumulative dose-dependent way [30], and it is presently unclear whether this effect is due to increased disease severity. In our study, no correlation between corticosteroid dose and the amount of intracellular ROS produced was observed. The patients had relatively low doses of corticosteroids (mean = 5 mg oral prednisone per day in treated patients) that are likely too low to affect the function of PMN. This could explain why no correlation with ROS levels was found. Moreover, other forms of immune suppressive drugs did neither seem to affect ROS production in the current setting.

Decreased neutrophil counts occur in SLE [31,32]. While this is partly due to autoantibodies, there is also evidence for direct effects on the bone marrow production of PMN. Bone marrow from SLE patients has decreased granulocyte-macrophage colony-forming units [31]–[33], and we show here that SLE patients have reduced numbers of newly released CD10^−^CD16^low^ neutrophils [6,7]. In agreement with earlier observations, these findings suggest an SLE-associated effect on the bone marrow with decreased release of new incompletely differentiated neutrophils. Hence, a decreased number of PMN will be found in the circulation, and with decreased numbers of PMN in the circulation, a prolonged half-life of the existing cells likely occur.

Another possibility is that the PMN phenotype in SLE patients is altered via an as-yet unidentified mechanism. The CD10 and CD16 molecules are normally stored intracellularly and can be rapidly mobilized to the cell surface upon activation [34]. Hence, an increased percentage of CD10^+^CD16^+^ cells and a corresponding decrease in CD10^−^CD16^low^ cells could reflect increased activation of PMN *in vivo* in SLE. In addition, the percentage of C5aR was decreased, indicating that PMN are activated in peripheral blood [35,36]. However, no increase in CD11b expression and corresponding decrease in CD62L were observed on SLE-PMN. Taken together, the observed altered PMN phenotype could be due to prolonged turnover of SLE-PMN in the circulation that gives rise to functional changes such as decreased ROS production and an atypical expression of surface markers.

## Conclusions

Our study shows an association between low ROS formation and disease severity in SLE. This is consistent with findings in other autoimmune disease, suggesting that a decrease in NADPH complex-mediated ROS production is a risk factor in autoimmunity. The phenotype observed in SLE-PMN could be due to aberrant production of leukocytes in the bone marrow and/or *in vivo* activation in the circulation. Future studies will illuminate the role of ROS formation and PMN in SLE and autoimmunity.

## Abbreviations

E. coli: *Escherichia coli*; NADPH: nicotinamide adenine dinucleotide phosphate-oxidase; NETs: Neutrophil extracellular traps; PMA: Phorbol 12-myristate 13-acetate; PMN: polymorphonuclear leukocytes; ROS: reactive oxygen species; SLE: systemic lupus erythematosus; SLEDAI-2 K: systemic lupus erythematosus disease activity index 2000; SLE-PMN: PMN from SLE patients; SLICC/ACR-DI: Systemic Lupus International Collaborative Clinics/American College of Rheumatology damage index.

## Competing interests

The authors declare that they have no competing interests.

## Authors’ contributions

ÅP, BG and ÅJ did the laboratory work. BG and AB gathered all clinical data. AB, TH, MH, SW, BG and ÅJ contributed to the design of the study and wrote the manuscript. All authors read and approved the final manuscript.
